# Thrombocytopenic, thromboembolic and haemorrhagic events following second dose with BNT162b2 and ChAdOx1: self-controlled case series analysis of the English national sentinel cohort

**DOI:** 10.1016/j.lanepe.2023.100681

**Published:** 2023-07-18

**Authors:** Mark Joy, Utkarsh Agrawal, Xuejuan Fan, Chris Robertson, Sneha N. Anand, Jose Ordonez-Mena, Rachel Byford, Rosalind Goudie, Gavin Jamie, Debasish Kar, John Williams, Gemma L. Marsden, Victoria Tzortziou-Brown, Sir Aziz Sheikh, F.D. Richard Hobbs, Simon de Lusignan

**Affiliations:** a Nuffield Department of Primary Care Health Sciences, University of Oxford, UK; b Department of Mathematics and Statistics, University of Strathclyde, Glasgow, UK; c Royal College of General Practitioners, London, UK; d Usher Institute, The University of Edinburgh, Edinburgh, UK; e Public Health Scotland, Glasgow, UK

**Keywords:** Epidemiology, COVID-19 vaccines, Vaccine safety

## Abstract

**Background:**

Thrombosis associated with thrombocytopenia was a matter of concern post first and second doses of BNT162b2 and ChAdOx1 COVID-19 vaccines. Therefore, it is important to investigate the risk of thrombocytopenic, thromboembolic and haemorrhagic events following a second dose of BNT162b2 and ChAdOx1 COVID-19 vaccines.

**Methods:**

We conducted a large-scale self-controlled case series analysis, using routine primary care data linked to hospital data, among 12.3 million individuals (16 years old and above) in England. We used the nationally representative Oxford-Royal College of General Practitioners (RCGP) sentinel network database with baseline and risk periods between 8th December 2020 and 11th June 2022. We included individuals who received two vaccine (primary) doses of the BNT162b2 mRNA (Pfizer-BioNTech) and two vaccine doses of ChAdOx1 nCoV-19 (Oxford-AstraZeneca) vaccines in our analyses. We carried out a self-controlled case series (SCCS) analysis for each outcome using a conditional Poisson regression model with an offset for the length of risk period. We reported the incidence rate ratios (IRRs) and 95% confidence intervals (CI) of thrombocytopenic, thromboembolic (including arterial and venous events) and haemorrhagic events, in the period of 0–27 days after receiving a second dose of BNT162b2 or ChAdOx1 vaccines compared to the baseline period (14 or more days prior to first dose, 28 or more days after the second dose and the time between 28 or more days after the first and 14 or more days prior to the second dose). We adjusted for a range of potential confounders, including age, sex, comorbidities and deprivation.

**Findings:**

Between December 8, 2020 and February 11, 2022, 6,306,306 individuals were vaccinated with two doses of BNT162b2 and 6,046,785 individuals were vaccinated with two doses of ChAdOx1. Compared to the baseline, our analysis show no increased risk of venous thromboembolic events (VTE) for both BNT162b2 (IRR 0.71, 95% CI: 0.65–0.770) and ChAdOx1 (IRR 0.91, 95% CI: 0.84–0.98); and similarly there was no increased risk for cerebral venous sinus thrombosis (CVST) for both BNT162b2 (IRR 0.87, 95% CI: 0.41–1.85) and ChAdOx1 (IRR 1.73, 95% CI: 0.82–3.68). We additionally report no difference in IRR for pulmonary embolus, and deep vein thrombosis, thrombocytopenia, including idiopathic thrombocytopenic purpura (ITP), and haemorrhagic events post second dose for both BNT162b2.

**Interpretation:**

Reassuringly, we found no associations between increased risk of thrombocytopenic, thromboembolic and haemorrhagic events post vaccination with second dose for either of these vaccines.

**Funding:**

Data and Connectivity: COVID-19 Vaccines Pharmacovigilance study.

**Research in context**

**Evidence before this study**

We searched PubMed, medRxiv and SSRN on June 27, 2022 for studies investigating serious COVID-19 outcomes following vaccination using the search terms “COVID-19 vaccine related thrombocytopenia (MeSH)”, “COVID-19 vaccine related thromboembolism (MeSH)”, “COVID-19 vaccine related haemorrhagic event (MeSH)”, “COVID-19 vaccines (MeSH)”, “COVID-19 vaccine related thrombocytopenia and thrombosis (MeSH)”, and “COVID-19 (MeSH)”. There was some evidence that the first dose of ChAdOx1 is associated with venous thrombotic events and thrombocytopenia. In parallel studies conducted in Scotland, ChAdOx1 was found to be associated with increased risks of idiopathic thrombocytopenic purpura, arterial thromboembolic and haemorrhagic events, while BNT162b2 was not associated with any risk. A study with a similar methodological was conducted in Wales, where both ChAdOx1 and BNT162b2 were found to be associated with an increased risk of thrombocytopenic, haemorrhagic, thromboembolic events.

**Added value of this study**

This study is one of the first to examine the risk of thrombocytopenic, thromboembolic and haemorrhagic events post-vaccination with two homologous doses of either ChAdOx1 or BNT162b2 COVID-19 vaccines in the adult population of England, UK. We found no association of increased risk of these events post second dose, which is slightly different to what was observed in Scotland and Wales. Although the healthcare systems and vaccines are similar (same brands and batches) in all the three nations (England, Scotland and Wales), the number of individuals in this study is much larger than the other two studies conducted in Scotland and Wales. The implementation of SNOMED CT in England provides more granular data which could be the reason for the difference.

**Implications of all the available evidence**

As the pandemic evolved, vaccination programmes and mitigations strategies evolved to prioritise those at risk of complications post COVID-19 infection and COVID-19 related hospitalisation and deaths. This English population based investigation did not find a safety signal following administration of a second dose of either of the ChAdOx1 and BNT162b2 COVID-19 vaccines. These data support the recommendation from the expert group that advised UK health departments that people should have the same brand of vaccine for their second dose, including for ChAdOx1.

## Introduction

The highly successful COVID-19 vaccination programme in England, started on 8th December 2020 with the roll out of the Pfizer-BioNTech BNT162b2 messenger ribonucleic acid (mRNA) vaccine. In January 2021 the Oxford-AstraZeneca ChAdOx1 nCoV-19 (ChAdOx1) recombinant technology vaccine was added to the programme.^1^^,^^2^ Initially the second dose of BNT162b2 was given three weeks after the first dose. However, this interval between doses was later modified to twelve weeks and then back to eight weeks.^3^^,^^4^ The roll out of vaccines was prioritised by the risk of hospitalisation and mortality from COVID-19,^5^ with the very oldest and care home staff given the BNT162b2, vaccine, with vaccination proceeding through progressively younger age groups. The administration of ChAdOx1 commenced a month later, starting with the oldest unvaccinated groups plus people in risk groups age 16–64 years. Subsequently, Moderna mRNA-1273, become the predominant vaccine for first and second dose, with additional BNT162b2 being administered in parallel.^6^

The first dose of ChAdOx1 was associated with venous thrombotic events and thrombocytopenia.^7^ The attributable risk was low with these events only noted after the first dose, and not with BNT162b2, with the observation period running up to mid-April 2021.^8^ It was also suggested that there might be an increased risk of stroke (including ischemic and haemorrhagic) following BNT162b2 vaccination.^9^ These findings were replicated in other studies,^10^, ^11^, ^12^ including a pooled UK analysis including our data.^13^ Both the European and UK medical regulatory bodies suggested that further investigation was required.^14^^,^^15^

Notwithstanding the initial reports of possible rare adverse events following the first dose of ChAdOx1 the UK expert group that advises the UK health departments, the Joint Committee on Vaccination and Immunisation (JCVI), recommended that for people over 30 years old, those who had the first dose of ChAdOx1 should have the same vaccine for their second dose. We thus carried out this study to explore whether the risk of thromboembolic (including arterial and venous events), thrombocytopenic and haemorrhagic events seen after the first dose of BNT162b2 and ChAdOx1 vaccines, were repeated after the second dose these vaccines. We used the nationally representative Oxford-Royal College of General Practitioners (RCGP) Research and Surveillance Centre (RSC) network database, the English primary care sentinel network database to conduct this study.^16^^,^^17^ The RSC is one of Europe's oldest sentinel systems and has been actively engaged in COVID-19 surveillance and vaccine effectiveness.^18^

## Methods

### Overview

We used the Oxford-Royal College of General Practitioners (RCGP) Research and Surveillance Centre (RSC) database, one of Europe's oldest sentinel networks that has a near real-time feed of primary care data and is nationally representative, covering around 32% of the English population (N > 19 million). Pseudonymisation was conducted using a National Health Service (NHS) Digital-approved process, allowing pseudonymised NHS numbers (unique national IDs) to link individual patient-level data to other datasets to supplement primary care data; these datasets included the second generation surveillance system for Pillar 1 (laboratory testing within NHS facilities) and Pillar 2 (community test facilities set up during the pandemic) COVID-19 infection results, the national immunisation management service for vaccine uptake, Hospital Episode Statistics for hospitalisation and intensive care unit admissions, and Office for National Statistics data for certificated cause of death.

We carried out a self-controlled case series (SCCS) using the RSC sentinel network database to study the association between the administration of second dose of BNT162b2 and ChAdOx1 COVID-19 vaccines and thromboembolic, thrombocytopenic and haemorrhagic events. This study is part of a UK-wide research collaboration with parallel studies completed in Wales^19^ and Scotland^20^; and part of a UK-wide collaboration.^21^ The base study cohort comprised of 12.3 million individuals in England, aged 16 years or older, with primary care records between 1st September 2019 and 11th June 2022. Individuals who received a second dose of either BNT162b2 or ChAdOx1 vaccines were eligible for the study.

An incident case was defined as the first event occurring in the period from when vaccination began in the UK (8th December 2020) until study end, with no prior thrombocytopenic, venous or arterial thromboembolic, or haemorrhagic clinical events since September 1st 2019.

Patients were followed up from December 8th 2020 to the earliest of the end of study or death. If a patient had more than one event of interest, only the first was used in the analysis.

### Study cohort

The study cohort is comprised of individuals aged 16 years old and over, registered with general practices in England (around 33%), and are recruited to be nationally representative.^16^ All the datasets are pseudonymised and linked to achieve a one-to-one match at individual level.^22^ Vaccine exposure data were collected from the primary care computerised medical record (CMR) system data and also from the National Immunisation Management System (NIMS). As the COVID-19 vaccine rollout was managed centrally the NIMS system data was used preferentially. This study is a complete case analysis and there was no missing data.

### Exposure

To analyse the associations, we considered an individual exposed from the day they received their second dose of either BNT162b2 or ChAdOx1 vaccine for up to 28 days. Person days at risk were reported for the time periods 0–6, 7–13, 14–20, and 21–27 days post second COVID-19 vaccine; we also reported overall events from the day of vaccination (zero) to within 28, i.e. 0–27, days.

### Outcomes

Our outcomes of interest were first clinical diagnosis of thromboembolic events, including venous thromboembolism (VTE), thrombocytopenia, including idiopathic thrombocytopenic purpura (ITP), arterial thrombosis and haemorrhagic events in primary or secondary care CMRs. We describe for each outcome of interest the number of events overall, then the number of person days in baseline, clearance and risk periods in person days. We then report the incident rate ratio (IRR) for each comparing baseline and risk periods. For VTE we report overall VTE events, then cerebral venous sinus thrombosis (CVST), pulmonary embolism (PE), and deep vein thrombosis (DVT). The International Classification of Diseases 10th revision (ICD-10) code for hospital outcomes and Systematised Nomenclature of Medicine (SNOMED) Clinical Terms (CT) were used to identify the events, and are included in a supplementary file. We adopted the same approach to using ICD-10 codes and primary care codes as used in our first dose study,^13^ and the parallel analyses in Wales and Scotland.

### Statistical analysis

**Fig. 1:**
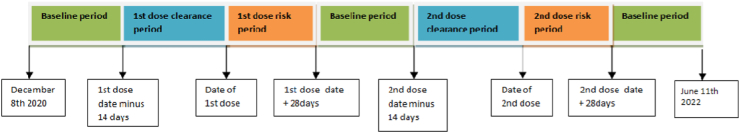
**Schematic presentationof the self-controlled case series including baseline, clearance and risk periods.**

The SCCS models for each outcome were estimated using a conditional Poisson regression model with an offset for the length of the risk period. IRR for each outcome, comparing risk periods to baseline periods were estimated using the SCCS model, adjusting for week (during the study period from 8th December 2020 to 11th June 2022). We are analysing events of interest after both doses so the complications and events after the first dose will be assigned to the relevant risk period in the modelling. In the same way, events after the second dose are assigned to the relevant post vaccination risk period. The clearance period is up to 14 days before either of the vaccination doses. The purpose of the clearance period is to adjust for the potential bias arising from unwell individuals not presenting for vaccination. The post vaccination risk periods occur 0–27 days after either of the vaccination dose and the remaining time is baseline period. The exposure term for first or second dose of each vaccination brand were included in the models: we defined the exposure risk intervals as 0–6, 7–13, 14–20 and 21–27 days post exposure date. A 14-day clearance period was used prior to the exposure date. The baseline period consisted of all remaining time in the period December 8th 2020–11th June 2022 (excluding clearance and risk periods, see Fig. 1).

### Ethical considerations

Data for this study were extracted from volunteer general practices who are part of the Oxford-RCGP RSC primary care sentinel network.^16^ Use of these data for this study was approved by the UK's Health Research Authority London Central Ethics Committee, reference No 21/HRA/2786/AM01, dated 15th June. This was a substantial amendment of an earlier application due to extension of the project (Integrated Research Application System (IRAS) ID 301740); Research Ethics Committee Reference 21/HRA/2786. Numerical values of five people or less were reported as “≤5” to avoid any risk of identification.

### Role of the funding source

The funders of the study had no role in study design, data collection, data analysis, data interpretation, or writing of the report.

## Results

This RSC English primary care sentinel network cohort held records of 12, 353, 091 individuals who had received two doses of COVID-19 vaccination, 6,306,306 (51.1%) of whom were vaccinated with two doses of BNT162b2 and the remaining 6,046,785 (48.9%) were vaccinated with two doses of ChAdOx1. Among individuals vaccinated with BNT162b2, a total of 8 and 104 CVST events were observed during the risk period (0–27 days post second dose of COVID-19 vaccine) and baseline period respectively with similar rate of events (2.0 per thousand person days). For ChAdOx1, the rate of events was slightly higher during the risk period (4.0 per thousand person days) in comparison to the baseline period (2.0 per thousand person days), with 10 and 102 events respectively. The rate of events for ITP was slightly higher for both the vaccines during the risk period (BNT162b2: n = 15, 3.5 per thousand person days & ChAdOx1: n = 18, 4.2 per thousand person days), in comparison to the baseline period (BNT162b2: n = 144, 2.0 per thousand person days & ChAdOx1: n = 147, 2.0 per thousand person days).

### Venous thromboembolic events (VTE)

We found no increase in the IRR of VTE events in any of the post second dose time intervals observed for both BNT162b2 (IRR 0.71, 95% CI: 0.65–0.77) and ChAdOx1 (IRR 0.91, 95% CI: 0.84–0.98). See Tables 1 and 2 for a description of events by each week post the second dose for BNT162b2 and ChAdOx1 respectively.

**Table 1:** Venous thromboembolic events for second dose BNT162b2 vaccine.

Time-period post vaccination	Number of events	Number of person days	Incidence rate ratio (95% confidence interval)
Venous thromboembolic events (VTE)			
Baseline	11,827	5,665,343	1.0
Clearance	248	154,701	0.61 (0.54–0.69)
0–6 days	142	77,889	0.68 (0.57–0.80)
7–13 days	154	76,740	0.75 (0.64–0.88)
14–20 days	127	75,966	0.63 (0.52–0.75)
21–27 days	154	75,202	0.77 (0.66–0.91)
0–27 days	577	305,797	0.71 (0.65–0.77)
Cerebral venous sinus thrombosis (CVST)			
Baseline	104	53,831	1.0
Clearance	0	1807	0 (NA)
0–6 days	≤5	910	1.29 (0.40–4.15)
7–13 days	≤5	910	0.87 (0.21–3.58)
14–20 days	≤5	910	0.44 (0.06–3.15)
21–27 days	≤5	904	0.90 (0.22–3.70)
0–27 days	8	3634	0.87 (0.41–1.85)
Pulmonary embolism (PE)			
Baseline	5873	2,736,333	1.0
Clearance	95	77,310	0.44 (0.36–0.54)
0–6 days	65	38,912	0.58 (0.45–0.75)
7–13 days	82	38,332	0.75 (0.60–0.94)
14–20 days	58	37,938	0.54 (0.41–0.70)
21–27 days	74	37,499	0.70 (0.56–0.89)
0–27 days	279	152,671	0.64 (0.57–0.73)
Deep vein thrombosis (DVT)			
Baseline	5970	2,939,986	1.0
Clearance	147	77,371	0.76 (0.64–0.89)
0–6 days	79	38,970	0.80 (0.64–1.0)
7–13 days	72	38,386	0.74 (0.59–0.94)
14–20 days	67	37,986	0.70 (0.55–0.90)
21–27 days	85	37,667	0.89 (0.72–1.1)
0–27 days	303	153,009	0.78 (0.69–0.90)

**Table 2:** Venous thromboembolic events for second dose ChAdOx1 vaccine.

Time-period post vaccination	Number of events	Number of person days	Incidence rate ratio (95% confidence interval)
Venous thromboembolic events (VTE)			
Baseline	11,381	5,566,675	1.0
Clearance	391	186,900	0.82 (0.73–0.91)
0–6 days	148	93,430	0.61 (0.52–0.72)
7–13 days	269	93,317	1.11 (0.98–1.27)
14–20 days	213	92,994	0.88 (0.77–1.0)
21–27 days	250	92,525	1.0 (0.91–1.18)
0–27 days	880	372,266	0.91 (0.84–0.98)
Cerebral venous sinus thrombosis (CVST)			
Baseline	102	55,344	1.0
Clearance	0	1316	0.0 (NA)
0–6 days	≤5	658	0.73 (0.09–5.41)
7–13 days	≤5	658	2.79 (0.94–8.17)
14–20 days	≤5	652	1.36 (0.31–5.80)
21–27 days	≤5	644	2.0 (0.60–6.73)
0–27 days	10	2612	1.73 (0.82–3.68)
Pulmonary embolism (PE)			
Baseline	5668	2,694,185	1.0
Clearance	172	91,014	0.72 (0.61–0.84)
0–6 days	74	45,489	0.62 (0.49–0.78)
7–13 days	126	45,418	1.1 (0.89–1.28)
14–20 days	101	45,254	0.86 (0.71–1.1)
21–27 days	106	44,954	0.92 (0.75–1.12)
0–27 days	407	181,115	0.87 (0.77–0.97)
Deep vein thrombosis (DVT)			
Baseline	5729	2,882,244	1.0
Clearance	219	96,236	0.90 (0.78–1.0)
0–6 days	75	48,116	0.61 (0.48–0.77)
7–13 days	146	48,084	1.17 (0.98–1.39)
14–20 days	110	47,922	0.88 (0.72–1.1)
21–27 days	141	47,764	1.12 (0.94–1.34)
0–27 days	472	191,886	0.94 (0.85–1.1)

We found similar number of events in the baseline periods for the specific VTE of interest, CVST, PE and DVT. However, the IRR across all individual observation periods was not significantly increased with either vaccine (Tables 1 and 2). Whilst many of the IRR were numerically higher for ChAdOx1 compared to BNT162b2, all the confidence intervals crossed parity. In the 27 days following a ChAdOx1 vaccination, we observed no CVST events in those aged 65 years and older; there were≤5 such events following a BNT162b2 vaccination.

### Thrombocytopenia

We did not find any association of thrombocytopenia following the second dose vaccination for both BNT162b2 (IRR 0.79, 95% CI: 0.64–0.98) and ChAdOx1 (IRR 1.47, 95% CI: 0.59–3.63). For both vaccines the baseline number of events and number of person days are very similar. As with VTE the numeric IRR for ChAdOx1 are greater than for BNT162b2 but again all the IRR 95% confidence intervals cross parity (Tables 3 and 4).

**Table 3:** Thrombocytopenic events for second dose BNT162b2 vaccine.

Time-period post vaccination	Number of events	Number of person days	Incidence rate ratio (95% confidence interval)
Thrombocytopenia			
Baseline	1873	929,459	1.0
Clearance	26	26,803	0.39 (0.27–0.59)
0–6 days	24	13,440	0.71 (0.47–1.07)
7–13 days	27	13,237	0.82 (0.56–1.20)
14–20 days	34	13,167	1.05 (0.74–1.48)
21–27 days	19	13,104	0.60 (0.38–0.94)
0–27 days	104	52,948	0.79 (0.64–0.98)
Idiopathic Thrombocytopenic purpura (ITP)			
Baseline	144	79,188	1.0
Clearance	≤5	2142	0.34 (0.10–1.07)
0–6 days	≤5	1071	0.89 (0.32–2.46)
7–13 days	≤5	1066	1.57 (0.71–3.46)
14–20 days	≤5	1064	1.73 (0.87–3.77)
21–27 days	≤5	1064	1.75 (0.88–3.86)
0–27 days	15	4265	1.26 (0.68–2.82)

**Table 4:** Thrombocytopenic events for second dose ChAdOx1 vaccine.

Time-period post vaccination	Number of events	Number of person days	Incidence rate ratio (95% confidence interval)
Thrombocytopenia			
Baseline	1786	929,694	1.0
Clearance	42	26,600	0.55 (0.16–1.79)
0–6 days	45	13,294	1.53 (0.54–4.35)
7–13 days	41	13,277	1.60 (0.57–4.53)
14–20 days	42	13,196	1.56 (0.57–4.49)
21–27 days	47	13,149	1.18 (0.36–3.84)
0–27 days	175	52,916	1.47 (0.59–3.63)
idiopathic thrombocytopenic purpura (ITP)			
Baseline	147	74,788	1.0
Clearance	≤5	2164	0.67 (0.22–3.69)
0–6 days	≤5	1085	0.90 (0.34–2.54)
7–13 days	≤5	1063	1.37 (0.43–4.41)
14–20 days	6	1054	2.74 (0.89–6.54)
21–27 days	≤5	1037	0.49 (0.07–3.53)
0–27 days	18	4239	1.38 (0.73–2.61)

Similarly, we did not find any significant increased incidence of ITP for both BNT162b2 (IRR 1.26, 95% CI: 0.68–2.82) and ChAdOx1 (IRR 1.38, 95% CI: 0.73–2.61) respectively (Tables 3 and 4). There were small (≤5) numbers of ITP events in the 0–27 days following either vaccination type for those aged 65 years and older.

### Arterial thromboembolic and haemorrhagic events

Finally, we looked at arterial thromboembolic and haemorrhagic events (Tables 5 and 6). There was no increase incidence of either in our post vaccination periods. For arterial thromboembolic events, the event number, and overall IRR in the first four weeks i.e. 0–27 days for BNT162b2 (IRR 0.96, 95% CI: 0.91–1.0) and ChAdOx1 (IRR 0.94, 95% CI: 0.89–0.98) COVID-19 vaccines were very similar including for each post vaccine observation period.

**Table 5:** Arterial thromboembolic and haemorrhagic events for second dose BNT162b2 vaccine.

Time-period post vaccination	Number of events	Number of person days	Incidence rate ratio (95% confidence interval)
Arterial thromboembolic events			
Baseline	30,576	15,194,492	1.0
Clearance	681	357,332	0.77 (0.71–0.83)
0–6 days	411	180,286	0.89 (0.81–0.99)
7–13 days	452	176,569	1.0 (0.92–1.10)
14–20 days	372	175,370	0.83 (0.75–0.93)
21–27 days	483	174,258	1.1 (1.0–1.20)
0–27 days	1718	706,483	0.96 (0.91–1.0)
Haemorrhagic events			
Baseline	2388	1,269,064	1.0
Clearance	72	32,345	1.1 (0.86–1.39)
0–6 days	29	16,215	0.88 (0.61–1.27)
7–13 days	27	16,111	0.82 (0.56–1.20)
14–20 days	31	16,010	0.95 (0.66–1.36)
21–27 days	32	15,916	0.99 (0.69–1.41)
0–27 days	119	64,252	0.91 (0.75–1.10)

**Table 6:** Arterial thromboembolic and haemorrhagic events for second dose ChAdOx1 vaccine.

Time-period post vaccination	Number of events	Number of person days	Incidence rate ratio (95% confidence interval)
Arterial thromboembolic events			
Baseline	29,270	14,658,015	1.0
Clearance	1114	534,716	0.81 (0.76–0.87)
0–6 days	617	267,257	0.89 (0.82–0.97)
7–13 days	700	266,878	1.0 (0.94–1.1)
14–20 days	710	266,243	1.0 (0.95–1.1)
21–27 days	564	265,198	0.82 (0.75–0.89)
0–27 days	2591	1,065,576	0.94 (0.89–0.98)
Haemorrhagic events			
Baseline	2322	1,256,052	1.0
Clearance	76	36,582	0.99 (0.78–1.26)
0–6 days	44	18,291	1.14 (0.84–1.56)
7–13 days	43	18,266	1.11 (0.81–1.52)
14–20 days	46	18,255	1.17 (0.87–1.59)
21–27 days	48	18,215	1.22 (0.91–1.64)
0–27 days	181	73,027	1.17 (0.98–1.38)

For haemorrhagic events the events in the baseline period were similar, across a similar number of person days (Tables 5 and 6). Although numerically different, there was no significant signal between BNT162b2 (IRR 0.91, 95% CI: 0.75–1.10) and ChAdOx1 (IRR 1.17, 95% CI: 0.98–1.38).

## Discussion

### Principal findings

In an SCCS study, including the records of over 12 million people, half (6.3 million) were vaccinated with two doses of BNT162b2 and the other half (6.1 million) vaccinated with ChAdOx1, we did not find a safety signal for our diagnoses of interest. There were 6344 (1457 VTE, 279 Thrombocytopenia events, 4308 Arterial thromboembolic events 300 Haemorrhagic events) individuals who experienced the event of interest during the risk period (0–27 days post second dose of BNT162b2 or ChAdOx1 COVID-19 vaccine). For some of the very rare events of interest, particularly CVST (n = 18, for both vaccines) and ITP (n = 33), the number of cases was very low (Table 1, Table 2, Table 3, Table 4), in the risk period post second dose. The findings in this study vindicate the decision of JCVI to continue to recommend the administration of a second dose of ChAdOx1.

### Comparison with the literature

Other studies have been similarly reassuring, these include a regional study from Spain,^23^ and a national study from Denmark.^24^ It also appears, though based on small numbers, that vaccination of people with previous CVST is safe.^25^ We note that the rate of CVST among the individuals who received ChAdOx1 vaccine was double that at baseline (IRR 1.73). However, there was a wide confidence interval crossing parity (95% CI: 0.82–3.68) so no statistically significant difference in rate. This lack of a safety signal contrasts with the signal detected after the first dose in younger adults where this was detected for CVST associated with thrombocytopenia following the first dose of ChAdOx1.^26^^,^^27^ Rates of arterial thrombosis and haemorrhage have also been found to be no higher post infection in other studies, including other UK studies.^28^

Compared to any of the previous studies, we have more follow-up time, have more individuals in the study cohort (i.e. more person-years) and are an ethnically more diverse cohort. Moreover, compared to the parallel studies performed in Scotland and Wales, the IRR of VTE events were no different, but with the smaller samples these studies were not able to estimate an IRR for CVST across the individual risk periods. We did not see any clustering of CVST cases in the 7–13 day risk period as was reported from Scottish data. The same applied to thrombocytopenia and ITP when we compare the equivalent risk periods.

### Strengths and limitations of the study

There are limitations to this study. Firstly, routine data are inevitably incomplete. Secondly, we could not access secondary care data in a timely way, which slowed-down our ability to report. A shortcoming is that our linked hospital data did not have platelet counts, and we had to rely on the recording of a thrombocytopenia diagnosis. Another shortcoming is relatively low quality of recording for venous thromboembolism, however this does not affect the outcome of this study. Another limitation is that we could not include related condition thrombosis and thrombocytopenia syndrome (TTS) as there is no single clinical code for it. Since TTS is a complex syndrome with competing definitions, it is particularly challenging to define the time windows and the acceptable temporal sequence for the co-occurrence of thrombosis and thrombocytopenia.

The likely under recording of long COVID reminds us that there can be gaps in data quality.^29^ Another limitation of this study is that we are only analysing the rate of events post vaccination with the second dose as our study was not set up to report on individuals who experienced similar events following the first dose of these vaccines. Finally, Scotland and Wales use the less granular Read terminology whilst England has move on to work with SNOMED CT; the latter is more granular allowing more specific diagnoses and symptom recording.^30^

Our strengths include the quality of data from RSC, which is a primary care sentinel network with pseudonymised data extracted either daily or twice-weekly and are part of national surveillance reports. Additionally, the occurrence of thrombosis and thrombocytopenia was extensively reported after the first dose of COVID-19 vaccine particularly ChAdOx1^7^, ^8^, ^9^, ^10^, ^11^, ^12^, ^13^, ^14^, ^15^ This study was part of a UK wide collaboration with long public health experience, and shared methodological approaches with similar studies conducted in Scotland and Wales.

### Implications of the findings

The failure to find any safety signal in the large study (n = 12.4 million) of people who have received two doses of either BNT162b2 or ChAdOx1is reassuring. Whilst this study is reassuring, analyses of larger populations and meta-analyses of studies is required to detect whether there is a safety signal.

### Call for further research

Internationally, we need to maintain the higher levels of digital maturity achieved during the pandemic and ensure these datasets are of the highest data quality.^31^ We should learn to work with entire national datasets, or with international data, especially to detect rare events. A limited primary care dataset of entire English national health data are available for research, with studies based on 46 million adults published, though not yet on second dose safety.^32^ There could be further research to investigate if the events of interest occurred following two doses of the different vaccine brands. Moreover, further research needs to be carried out to analyse the risk of mortality among vaccinated individuals with these events.

### Conclusions

Our SCCS investigation of Thromboembolic, thrombocytopenic and haemorrhagic events following second dose BNT162b2 and ChAdOx1 COVID-19 vaccination in England did not detect any signal, across over 12 million people who had received a second dose of either vaccine. Limitations of our study are that we used routine data, albeit from a sentinel network and linked to hospital data did not include immediate platelet results around their time of admission. In conclusion, this study shows that it is safe to have the same vaccine twice, supporting the recommendation from UK health departments that individuals should have the same brand of vaccine for their second dose.

## Contributors

SdeL and MJ conceived the English version of this DaCVaP study. MJ conducted the analysis, created methods and results tables. SdeL drafted the paper. UA edited the paper. CR advised on methodology for analysis. XF, RB, RG extracted and provided data tables for statistical analysis. GJ, DK, JW created clinical variables associated with the study. All authors were part of the DaCVaP study reviewed the findings and commented on the final manuscript.

## Data sharing statement

The data used in this study are sensitive and will not be made publicly available. All codes used in this study are available upon request.

## Declaration of interests

SdeL is Director of the Oxford-Royal College of General Practitioners (RCGP) Research and Surveillance Centre (RSC). SdeL has received funding for vaccine related research from AstraZeneca, GSK, Sanofi, Seqirus and Takeda, and been members of advisory boards for AstraZeneca, Sanofi and Seqirus all through his university. The AstraZeneca studies include ATTEST a study of Thrombotic Thrombocytopenia (SdeL is PI and MJ and FDRH Co–I). JMOM has previously worked on vaccine related project funded by AstraZeneca. GJ has previously presented to healthcare professionals on heart failure treatment which was funded by AstraZeneca. FDRH has previously received consultation fees on MAbs and vaccines from AstraZeneca and Pfizer. FDRH has previously received fees for presenting at Cardio-vascular diseases and COVID-19 related events organised by AstraZeneca, Pfizer, Boehringer Ingelheim and Bristol Myers Squibb. AS and CR are members of the Scottish Government's CMO COVID-19 Advisory Group and its Standing Committee on Pandemics. AS was a member of AstraZeneca's Thrombotic Thrombocytopenic Taskforce and all of his roles are unremunerated. CR is a member of the Scientific Pandemic Influenza Group on Modelling, Medicines and Healthcare products Regulatory Agency Vaccine Benefit and Risk Working Group. CR also received support from Public Health Scotland, MRC and CSO. VTB is member of various NHSE advisory boards on issues related to NHS workforce and workload, UK Health Alliance on Climate Change on behalf of RCGP and Our Future Health advisory board on behalf of RCGP. VTB held leadership positions as RCGP Vice Chair, BMA member, FSEM member, FSRH member, SAPC member and exec member (from July 2022), Founding participant of Health Foundation Q initiative, Member of the Allocations Steering Group-NHS England and Improvement, Member of NIHR Primary Care Strategy group, Council member and Senior Founding Fellow of Faculty of Medical Leadership and Management, NED Turning Point. VTB has previously received expenses for RCGP events, Nuffield Trust events and HSJ events. All other authors declare no competing interests.
